# Using a quality improvement approach to improve reporting of malaria deaths in Namutumba District, Eastern Uganda, 2022–2023

**DOI:** 10.1371/journal.pgph.0003324

**Published:** 2025-06-02

**Authors:** Marie Gorreti Zalwango, Richard Migisha, Benon Kwesiga, Lilian Bulage, Daniel Kadobera, Alex Riolexus Ario

**Affiliations:** Uganda Public Health Fellowship Program, Uganda National Institute of Public Health, Kampala, Uganda; Jawaharlal Nehru Medical College, INDIA

## Abstract

**Background:**

Reporting malaria deaths is critical for assessing prevention and case management interventions. In Uganda, malaria mortality is recorded in inpatient registers and reported through weekly and monthly surveillance reports. During a data quality assessment in Namutumba District in October 2022, we found more malaria deaths in health facility registers than were reported. We conducted a continuous quality improvement initiative to improve the accuracy of reported malaria deaths in Namutumba District.

**Methods:**

We purposively selected 2 high-level health centers (HC) in Namutumba District that reported malaria deaths during September 2021–October 2022. We formed quality improvement teams (QIT) comprising clinical and statistical staff at the HC. We conducted brainstorming sessions with QITs to identify challenges with reporting malaria deaths, prioritized areas for improvement, and conducted root cause analysis. Using the plan, do, study, act (PDSA) cycle, we identified change ideas to address root causes.

**Interventions:**

Challenges included knowledge gaps on malaria death definitions, lack of consequences for failing to document deaths, and unclear guidance on how to document deaths. Sustainable interventions identified included continuous medical education on malaria death definition, one-on-one mentorship of staff on documentation in inpatient registers, and weekly verification of inpatient register data, all implemented during November 2022–February 2023.

**Results:**

Of the 36 malaria deaths that occurred during the baseline period (September 2021–October 2022), 25 (69%) were included in the weekly report, and four (11%) in the monthly report. Following the intervention implementation, all 7 malaria deaths recorded at the 2 health facilities during November 2022–February 2023 were reported in the weekly and monthly reports.

**Conclusion:**

Continuous medical education, supervision and mentoring of HC staff, and clear and comprehensive guidance on documenting malaria deaths contributed to the improvement in malaria death reporting for HCs in Namutumba District. Consistence in implementation of these improvement activities could enable accurate planning and resource allocation for malaria control strategies.

## Introduction

Malaria is one of the leading causes of mortality globally, responsible for 608,000 deaths in 2022 The vast majority (96%) of the reported deaths occur in 29 sub-Saharan Africa countries Uganda inclusive. Uganda ranks eighth globally among countries with the highest burden of malaria deaths. It causes 20–30 deaths weekly and around 1,300 annually, as per the Uganda Health Management Information System (HMIS) [1]. Due to challenges in reporting malaria deaths, the HMIS-reported numbers have been adjusted for underreporting, resulting in an estimated 19,600 deaths annually, according to the World Malaria Report [2].

Based on findings from a community study conducted in Namutumba District, several community malaria deaths were documented at health facilities but not recorded in the health information system due to deaths occurring before reaching high-level facilities [3]. Subsequently, we conducted a data quality assessment (DQA) from September 2021 to October 2022 with the aim of determining the accuracy of health facility malaria deaths data. By comparing registers and client cards to surveillance reports (HMIS 033b and HMIS 108) through the District Health Information System version 2 (DHIS2), the DQA revealed a discrepancy. Out of 36 malaria deaths recorded, 25 (69%) were included in the weekly report, and 4 (11%) in the monthly report, indicating an inconsistency in reporting between registers and surveillance reports.

The low reporting of actual numbers of malaria as the cause of deaths due to various challenges contribute to underestimation of the true magnitude of malaria deaths. Without accurate reporting of malaria mortality data at the district and national levels, planning for effective interventions to prevent malaria deaths remains insufficient, posing a challenge to the goal of malaria eradication by 2030. This initiative focused on improving the accuracy of reported malaria deaths in selected health facilities in Namutumba District during September 2021–October 2022, to provide a case study for improvement in other health facilities and districts in Uganda.

### Rationale for quality improvement approach

Quality improvement is the combined and unceasing efforts of everyone to make the changes that will lead to better patient outcomes (health), better system performance (care) and better professional development[4]. Quality improvement approaches are based on the principle that there is an opportunity for improvement in every process and on every occasion and include small samples, frequent changes in interventions, and adoption of new strategies that appear to be effective for a particular setting. According to the Institute of Medicine (IOM) report, majority of medical errors result from faulty systems and inefficient processes not individuals. This makes other improvement approaches like staff training, enhanced surveillance techniques among others ineffective for such complex systems[5–7].

## Methods

### Project implementation setting

We conducted the quality improvement project in Namutumba District based on the previous low number of malaria deaths reported as per findings from the DQA. Namutumba District is located in the eastern part of Uganda, a region highly endemic for malaria [8,9]. The district is made up of 2 constituencies and 20 sub-counties and has a total population of approximately 320,000 people. The population is served by 2 private hospitals, 1 public health center (HC) IV, seven HC IIIs, and 25 HC IIs [10]. However, due to the low economic status of the residents in the district, majority of the patients utilize services at the HC IV which serves as the district referral health unit beyond which patients are referred to higher facilities outside the district for further management. We conducted the project in two high-level HCs in Namutumba District because of their high capacity for severe malaria admissions and the high likelihood for malaria deaths. These were Namutumba HC III and Nsinze HC IV.

### District administration engagement

Meetings were held with members of the district health team (DHT) specifically the district health officer, the assistant district health officer maternal and child health, the biostatistician and the malaria focal person in order to discuss the existing challenges and the proposed strategies for improvement for administrative clearance and support during project implementation and to ensure sustainability of the project achievements.

### Project implementation design

The quality improvement model used for our project focuses on 3 fundamental questions. What are we trying to accomplish? How will we know if a change is an improvement? and what changes can we make that will result in an improvement? Building on this information, we rejuvenated the Quality Improvement Team (QIT) for the selected facilities. This step was followed by collection of baseline data and we supported the teams to identify their problems and define their improvement objective. We further identified measures and changes to be made for improvement and implemented them using the Plan Do Study Act (PDSA) model.

### Plan Do Study Act (PDSA) model

Quality improvement projects and studies aimed at making positive changes in health care processes to effecting favorable outcomes can use the Plan-Do-Study-Act (PDSA) model. This is a method that has been widely used by the Institute for Healthcare Improvement for rapid cycle improvement. One of the unique features of this model is the cyclical nature of impacting and assessing change, most effectively accomplished through small and frequent PDSAs[11]. The purpose of PDSA quality improvement efforts is to establish a functional or causal relationship between changes in processes (specifically behaviors and capabilities) and outcomes. Langley and colleagues proposed three questions before using the PDSA cycles: (1) What is the goal of the project? (2) How will it be known whether the goal was reached? and (3) What will be done to reach the goal? The PDSA cycle starts with determining the nature and scope of the problem, what changes can and should be made, a plan for a specific change, who should be involved, what should be measured to understand the impact of change, and where the strategy will be targeted. Change is then implemented and data and information are collected. Results from the implementation study are assessed and interpreted by reviewing several key measurements that indicate success or failure. Lastly, action is taken on the results by implementing the change or beginning the process again[5,6,12,13].

### Continuous quality improvement team formation

We reconstituted the QIT at each facility to coordinate the implementation of project activities. The teams constituted the malaria focal person, in-charge, health facility QI focal person, records assistant, and two clinicians at the outpatient clinic and inpatient ward. The roles of the QIT were as follows: 1) Team lead – responsible for assigning roles and adding a voice where it was required. Members on the project were asked to appoint their own leader and change when need arise. 2) Mentor – responsible for guiding the team in carrying out certain roles, and provided mentorship to the team members. 3) Data person – responsible for data collection and analysis of the collected data. 4) Support personnel – provided information or any additional assistance required to conduct the project.

### Baseline data collection

We analyzed health facility data for Nsinze HC IV and Namutumba HC III for the period of September 2021 to October 2022 to serve as our baseline for malaria deaths reporting. This involved review of the HMIS data for the HMIS 033b weekly surveillance report and the HMIS 105 monthly report, review of client cards for all admitted patients, review of the inpatient register and the outpatient register for cases that might have died before admission. This information helped the team to identify gaps in data capture and reporting. Using a score of 0–5 on the spider graph (5 being the highest ranking), the identified gaps were prioritized for problem analysis.

### Process mapping and gap identification

We reviewed the process of data capture in the primary data tools, data collection and data entry into the electronic HMIS system through brain storming sessions with the facility teams. For each health facility, one brain storming session comprising of 10 team members from the various departments was held. The team members included: The health facility in charges, the district malaria focal person, the health information assistant, unit heads (Outpatient, Inpatient and special clinics), a nurse, a midwife, a clinician and a village health team member attached to the health facility. This was done through process mapping to identify the challenges and gaps in data capture and reporting from the point of patient entry into the facility to the point when the data generated is submitted to the national database. Areas of focus during process mapping included: data entry into the primary health facility registers, data abstraction from the registers, completion of hard copy HMIS reports and data entry from the hard copies to the health management information system. Through the discussions, gaps under each area were documented in excel and charted on a spider graph for prioritization of the area with the most gaps. We further assessed the health facility performance against the reporting attributes of timeliness, accuracy, reliability and completeness. For each attribute, the performance percentage was obtained through computing data reported in the national HMIS system as the numerator and data abstracted from the health facility registers to as the numerator multiplied by 100.

### Problem analysis and root cause identification

Following prioritization of the problem, the QIT did problem analysis for the identified gaps to identify the root causes for low reporting for malaria deaths using the ‘five whys’ technique. For each of the problems, the team exhausted the possible causes until when the root cause was reached. These findings were displayed using a fish bone diagram.

### Goal refinement and change idea identification

Following problem identification, the QIT refined the goal for the project which was a clear statement of the intended improvement and how it would be measured. The goal had the following attributes: specific, measurable, achievable, realistic and time bound. The QIT proposed changes/interventions to the identified problems. Teams identified the different change ideas that would bring about the desired change. These change ideas were organized into groups, each of which represents a similar notion or approach to change, or change concept. We then ranked the proposed changes.

### Intervention tools

Based on the proposed changes, we identified tools required to implement the suggested changes. We focused on tools that were cheap and readily available to ensure sustainability of the project.

### Intervention monitoring and evaluation

We used the PDSA cycles to plan and test the identified interventions. Through the weekly and monthly data collection, analysis and interpretation, the team was able to assess each implemented intervention for a positive change. The plan was to maintain interventions that improved reporting for malaria deaths, while interventions that did not cause change were dropped. The final evaluation was done in March 2023 to ascertain whether there was an improvement in the reporting rates of malaria deaths.

### Ethical considerations

We obtained administrative clearance from Namutumba District Health Office and the in charges for the selected health facilities. As part of the routine Quality Improvement (QI) processes, we conducted brainstorming sessions with health facility staff to identify gaps and generate change ideas for improving the reporting of malaria deaths. These sessions are integral to standard QI practices and did not involve formal research procedures requiring IRB approval. We obtained written informed consent from all the respondents who took part in the activity. They indicated their consent by checking an appropriate box for consent before proceeding with the interviews. Participants were assured that their participation was voluntary and that there would be no negative consequences for declining or withdrawing from the activity. Data collected did not contain any individual personal identifiers and information was stored in password-protected computers, which were inaccessible by anyone outside the project. Additional data used was obtained from routinely collected malaria surveillance data in the health facility registers and the DHIS2, which is publicly available for analysis and use in informing public health interventions. All were aggregated with no individual identifiers included. This activity was reviewed by US Centers for Disease Control and Prevention (CDC) and was conducted consistent with applicable federal law and CDC policy.§ §See, e.g., 45 C.F.R. part 46, 21 C.F.R. part 56; 42 U.S.C. §241(d); 5 U.S.C. §552a; 44 U.S.C. §3501 et seq. This determination was made because the project aimed to address a public health problem and had the primary intent of public health practice.

## Results

### Baseline findings

During the baseline period reviewed, Namutumba HC III recorded 4 malaria deaths. Only 50% (2/4) of the confirmed malaria deaths were reported in the monthly HMIS 108 report while 100% (4/4) were reported in the weekly surveillance report. For Nsinze HC IV, 32 malaria deaths were recorded in the period of September 2021-October 2022. Findings from the baseline data collection revealed that only 19% (6/32) confirmed malaria deaths were reported as in the monthly surveillance report while 81% (26/32) were reported in the weekly surveillance reports. We noted that HMIS 033b was collected from both out-patient and in-patient registers while the HMIS 108 report was collected from only the in-patient register for both health facilities as opposed to utilization of the inpatient register as the official data source for malaria mortality.

### Process mapping and gap identification

**Fig 1:**
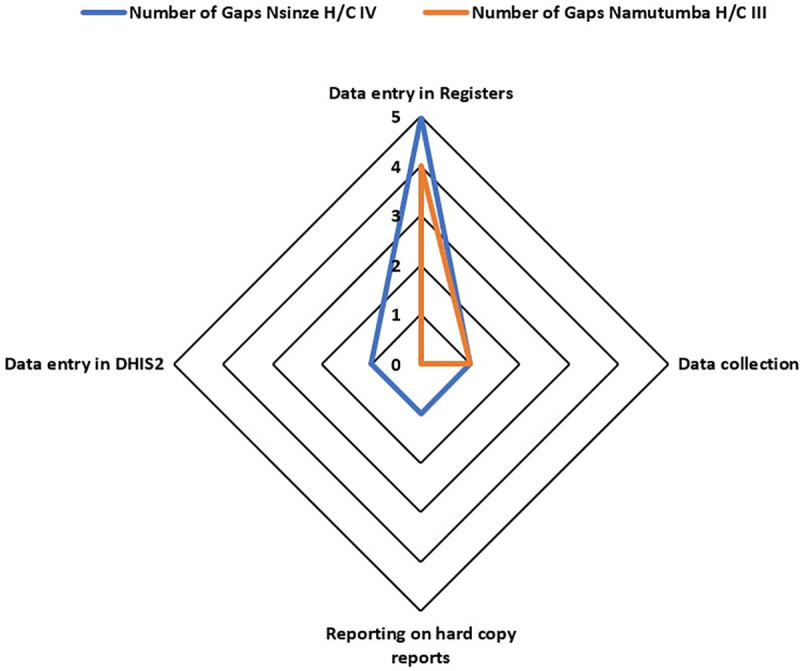
Number of gaps identified during the process mapping activity, Namutumba District, Eastern Uganda, September 2021–October 2022.

**Fig 2:**
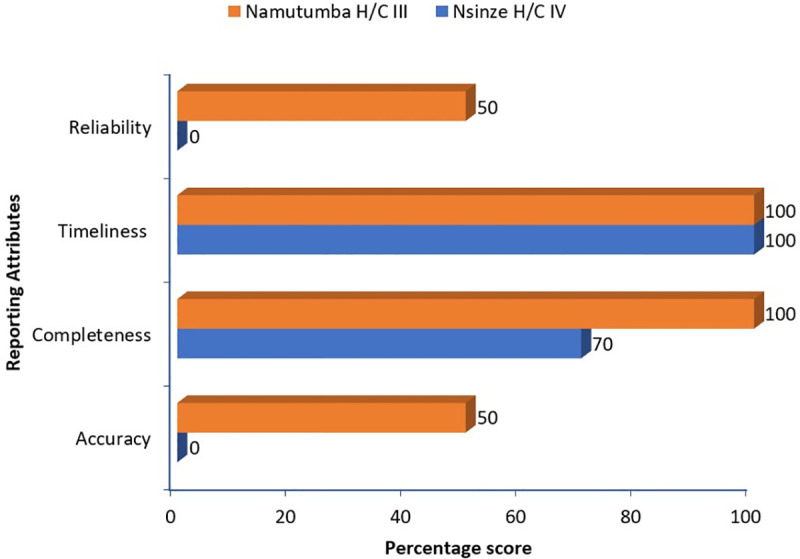
Performance of Nsinze H/C IV and Namutumba H/C III against the reporting attributes, Namutumba District, Eastern Uganda, October 2022.

Following review of the reporting system for malaria deaths, gaps were identified at the steps of data entry in the primary registers and data compilation from the registers. Other steps like data entry on hard copy reports and entry of data in the electronic HMIS had no gaps identified (Fig 1). This informed our choice to focus on data entry into the inpatient register for root cause analysis since this is the primary source document for malaria deaths data. On assessment of reporting attributes, Nsinze H/C IV, the highest level of care in the district scored 0% on reliability and accuracy of data and scored 70% on completeness; way lower than Namutumba H/C III (Fig 2).

### Root causes affecting entry of malaria deaths data in the in-patient registers for Namutumba District, September 2021–October 2022

**Fig 3:**
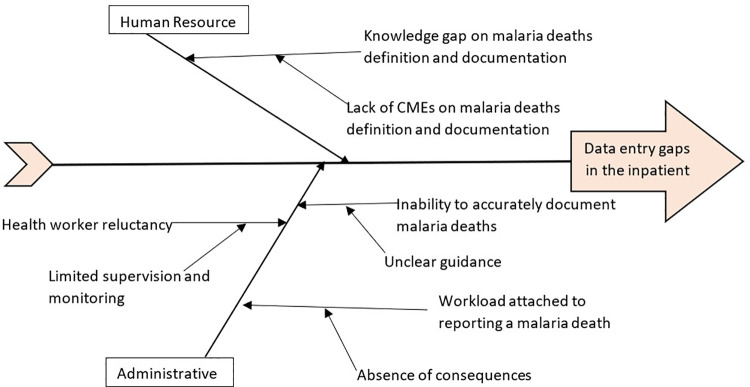
Root causes for the gaps identified during the problem identification at the health facilities in Namutumba District, Eastern Uganda, September 2021—October 2022.

As depicted in Fig 3 below, the root cause of failure to enter malaria death data included:

#### Lack of continuous medical education on malaria deaths definition and documentation .

Insufficient continuous medical education (CMEs) on the definition and documentation of malaria deaths led to health workers documenting complications rather than malaria as the cause of death in primary registers, despite malaria being the primary diagnosis. The knowledge gap resulting from the lack of CMEs contributed to low documentation and reporting of malaria deaths.

#### Absence of consequences for non-reporting of malaria deaths.

The absence of consequences for non-reporting of malaria deaths led to reluctance among health workers to document such deaths, as it involved additional paperwork, including filling out medical certificates of cause of death and entering data into the DHIS2.

#### Limited supervision and monitoring performance.

Limited supervision and monitoring performance of health workers contributed to staff reluctance in accurately documenting and reporting malaria deaths.

#### Unclear guidance on malaria death documentation.

Severe malaria deaths are expected to be recorded in the in-patient register; however, deaths occurring at health facility gates, outpatient department, and on the facility premises after referral were often not documented or recorded in the outpatient register. This inconsistency resulted in discrepancies between weekly and monthly reports, as the monthly report relies on both inpatient and outpatient registers. Similar challenges arose for deaths occurring on the way to the health facility or at home, as there was no clear guidance on documenting such deaths happening outside the inpatient wards and in the community. Furthermore, the reporting tools lack guidance on what constitutes a malaria death.

Following the brainstorming exercise, a range of change interventions were identified for improving reporting of malaria death data. These included:

a) Conducting regular CMEs on malaria death definition, recording, and reporting
b) Conducting CMEs to guide on the documentation of death on arrival and after referral
c) Conducting one on one mentorship on malaria deaths documentation
d) Establishment of focal persons to ensure timely update of malaria deaths in the IPD register
e) Conducting weekly malaria death data reviews
f) Strengthening the system of support supervision and monitoring

The change interventions were then turned into activities with clear indicators and means of verification and responsible persons identified for each activity (Table 1)

**Table 1:** Quality improvement interventions and indicators.

Intervention	Activity Description	Responsible Person	Indicator	Measure of Success	Means of verification
Conducting regular CMEs on malaria death definition, recording, and reporting	Bi-monthly CMEs on malaria deaths definition, documentation and reporting. Using a CME schedule, notification was made to all health facility team by in charges to ensure attendance. A didactic approach was used aided by power point presentations and use of flip charts.	Clinician responsible for CMEs	Proportion of health facility staff with knowledge of malaria death definition, recording, and reporting.	100%	Inpatient register
Conducting CMEs to guide on the documentation of death on arrival and after referral	The QIT discussed and agreed on the best way to document malaria death on arrival and after referral. Since there was no clear guidance, the team agreed that malaria deaths on arrival with positive malaria results and those dying in the resuscitation room would be documented in a special way in the inpatient register clearly indicating the place of deaths in the comment section. Patients dying on the health facility premised following referral would have their outcome changed from referred to Died and captured as a health facility death. These resolutions were provided to the rest of the team during the bi-weekly CMEs to ensure implementation by every staff member.	Clinician responsible for CMEs	Proportion of malaria deaths at the facility documented in the inpatient register.	100%	Inpatient register
Conducting one on one mentorship on malaria deaths documentation	Mentorship of clinicians (medical officers, nurses and midwives) and medical records staff on required data parameters for malaria deaths documentation	Clinician responsible for CMEs	% of health workers with competency to document malaria deaths completely and accurately	100%	Inpatient register
Establishment of focal persons to ensure timely update of malaria deaths in the IPD register	Assigned focal persons reviewed the registers after recording a death to ensure completeness and accurateness of documentation	Malaria Focal Person	Proportion of malaria deaths at the facility documented completely and accurately in the inpatient register.	100%	Inpatient register
Conducting weekly malaria death data reviews	Weekly review of inpatient client cards and Outpatient registers to ensure that all malaria deaths were documented in the inpatient register.	Quality Improvement Team	Proportion of malaria deaths at the facility documented in the inpatient register.	100%	Inpatient register
Strengthening the system of support supervision and monitoring	Weekly review of the inpatient registers to ensure that all malaria deaths recorded have medical certificates of cause of death filled and filed	Health Facility In charges	% malaria deaths reported in HMIS033b	100%	HMIS 033b report Inpatient register

### Final evaluation of the quality improvement project

**Fig 4:**
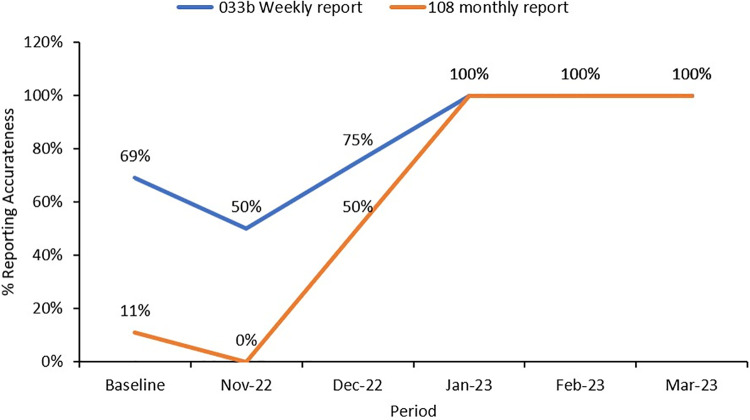
Changes in malaria mortality reporting for the health facilities in Namutumba District, Eastern Uganda, November 2022—March 2023.

Following implementation of QI project in Namutumba District, there was an improvement in the reporting of malaria deaths from 69% for the weekly report and 11% for the monthly report to 100% during the period of November 2022 to March 2023 with all the seven malaria deaths recorded and reported in the weekly and monthly reports. All the seven malaria deaths that happened in the health facilities were appropriately documented in the primary source document (inpatient register) and reported in the weekly surveillance and monthly reports for all the 5 months of project implementation (Fig 4). There was further an improvement in the reporting attributes scores; all health facilities scored 100% on the 4 reporting attributes assessed.

## Discussion

The project identified gaps and challenges in reporting malaria deaths, leading to low rates in both weekly and monthly surveillance reports. Contributing factors included: the absence of Continuous Medical Education (CME) on malaria deaths, limited supervision, lack of consequences for non-reporting, and unclear guidance on documenting deaths in specific scenarios. After implementing proposed changes, there was an improvement in reporting malaria deaths, enhancing data quality at health facility, district, and national levels. This improvement could positively impact planning efforts by various stakeholders.

The study revealed a knowledge gap among health workers regarding the definition of malaria death due to the lack of CME on this topic. This knowledge gap resulted in misclassification and the subsequent underestimation of the burden of malaria deaths. According to the district health information system version 2 (DHIS2), a malaria death is defined as any death with a positive malaria test in a reporting health facility or community [14,15]. Severe malaria that causes deaths presents with numerous complications like anaemia, respiratory failure, impaired consciousness and renal failure [16]. This complexity contributes to potential misclassification, particularly in the presence of a knowledge gap on the case definition of malaria death. Similar challenges have been observed in India, where a study highlighted the difficulty in defining and recording malaria deaths, because individuals with malaria may also suffer from other illnesses concurrently or in quick succession [17]. Regular CMEs are essential to ensure that all attending clinicians comprehend the accurate case definition for malaria deaths, facilitating accurate documentation and reporting.

Our study highlighted the lack of clear guidance on the documentation of malaria deaths upon arrival at the facility and deaths at the health facility premises after referral. As data on malaria deaths is extracted from the inpatient register (HMIS 075) and reported in the HMIS 108 report (Inpatient report), cases that do not reach the inpatient unit are often missed. Deaths on arrival might be recorded in the outpatient registers and included in the weekly surveillance report from the outpatient register, leading to inconsistencies between the HMIS 033b and HMIS 108 reports. The US Presidential Malaria Initiative (PMI), Roll Back Malaria (RBM) and Measure Evaluation have provided guidance for Evaluating the Impact of National Malaria Control Programs in Highly Endemic Countries. They highlight challenges in malaria-specific mortality surveillance and recommend using multiple data sources to compliment limitation from each method. Recommended sources in addition to the health information system include use of verbal autopsies, health and demographic surveillance systems (HDSS), civil registration and vital statistics systems and lastly the use of all child mortality as an impact indicator [18].

The study further revealed unclear guidance on documentation and recording of community malaria deaths. According to the NMCD, community/village health teams (VHTs) should follow up cases at home to ensure treatment compliance and recovery and to report any deaths due to malaria at home[14]. VHTs are required to report their data to health facilities quarterly; however, very few of them do report citing numerous challenges, including lack of transport, since in Uganda they are volunteers with no pay and with little supervision[19–21]. Scholars from other countries have suggested monetary facilitation and supervision to ensure they execute their roles[19,22]. Moreover, the quarterly reporting system lacks timeliness for informing interventions. As such, exploring more frequent reporting mechanisms could enhance the capture of accurate and up-to-date information. Establishing clear guidance on the optimal reporting of community malaria deaths and issuing an official memo to all districts on this matter could ensure that reported malaria death numbers accurately reflect the burden of malaria deaths, and facilitating effective planning.

Our study revealed staff reluctance to document malaria deaths due to the additional workload involved in reporting, such as completing the medical certificate of cause of death. In a study to evaluate the impact of system failures on health workers, researchers noted that a failure in the complex system where health workers work from causes physical and emotional burnout which may manifests as decreased production and lack of motivation. Additionally, in another study it was highlighted that the health workforce is not keeping up with the population growth and the epidemiologic and demographic trends of diseases due to the insufficient funding for the health sector and poor human resource management which impends recruitment and retention of health workers[23]. A system approach to existing challenges affecting health workers was proposed for improved performance[24]. Similarly, in our setting, addressing workload challenges by encouraging teamwork coupled with enhanced supervision and mentorship, could improve health worker productivity and ensure accurate reporting for effective planning of required interventions.

The lack of supervision and mentorship of health facility staff to ensure delivery of services as required was highlighted as a major challenge to reporting. In Uganda, health leadership and governance are primarily structured around a decentralized system where district local governments play a key role in managing healthcare delivery; the central government structure (Ministry of Health) sets standards and guidelines and focuses on policy development and oversight. This gives the district absolute powers for management of district resources including human resources for health. A study in Uganda highlighted that health workers are skilled, competent, and generally have a positive attitude to work, however, their performance is affected by the lack of performance management plans, unclear performance indicators, lack of monitoring for work schedules, and the widespread political interference and nepotism in the district health sector management[25]. We recommend streamlining district health sector management to address similar challenges and setting clear performance indicators not only for staff but also for district health leadership. Additionally, the Ministry of Health should be assigned as a regulatory authority that monitors health indicators’ performance, enforces compliance, and intervenes as necessary to address failures in achieving established goals. This could improve health sector efficiency and the eventual improvement in reporting across all indicators.

### Study limitations

Our study faced limitations regarding the timely reporting of community malaria deaths due to a lack of guidance from the Ministry of Health on capturing these data at health facilities on a weekly and monthly basis. This limitation could have led to an underestimation of malaria deaths in Namutumba District. However, we minimized this limitation by strengthening the documentation of all malaria deaths occurring on arrival and immediately after referral from the health facility.

## Conclusion

There was improvement in malaria mortality reporting for Nsinze HC IV and Namutumba HC III during and after the implementation of the quality improvement project. Changes that contributed to this improvement included: continuous medical education, supervision and monitoring performance of health facility staff, clear and comprehensive guidance on documentation of malaria deaths and weekly data reviews. Ensuring regular CMEs, data review, support supervision, and comprehensive guidance on documenting malaria deaths could enhance reporting for malaria deaths could reinforce efforts to achieve the malaria eradication target by 2030. Additionally, future studies to identify challenges in all-cause mortality reporting and interventions for improved all-cause mortality data benchmarking on the lessons learnt from this project could aiding targeted public health interventions for general premature mortality prevention.
